# Detrimental and Beneficial Effect of Autophagy and a Potential Therapeutic Target after Ischemic Stroke

**DOI:** 10.1155/2020/8372647

**Published:** 2020-09-23

**Authors:** Meng Wang, Hangil Lee, Kenneth Elkin, Redina Bardhi, Longfei Guan, Ankush Chandra, Xiaokun Geng, Yuchuan Ding

**Affiliations:** ^1^China-America Institute of Neuroscience, Beijing Luhe Hospital, Capital Medical University, Beijing, China; ^2^Department of Neurology, Beijing Luhe Hospital, Capital Medical University, Beijing, China; ^3^Department of Neurosurgery, Wayne State University School of Medicine, Detroit, MI, USA; ^4^Department of Research & Development Center, John D. Dingell VA Medical Center, Detroit, MI, USA

## Abstract

Autophagy, a physiologic mechanism that promotes energy recycling and orderly degradation through self-regulated disassembly of cellular components, helps maintain homeostasis. A series of evidences suggest that autophagy is activated as a response to ischemia and has been well-characterized as a therapeutic target. However, the role of autophagy after ischemia remains controversial. Activated-autophagy can remove necrotic substances against ischemic injury to promote cell survival. On the contrary, activation of autophagy may further aggravate ischemic injury, causing cell death. Therefore, the present review will examine the current understanding of the precise mechanism and role of autophagy in ischemia and recent neuroprotective therapies on autophagy, drug therapies, and nondrug therapies, including electroacupuncture (EA).

## 1. Introduction

Globally, stroke is the leading cause of disability and presents a great financial burden due to its “3H” effects: high disability, high morbidity, and high mortality [1, 2]. Acute ischemic stroke dominates among the spectrum of stroke disorders and leads to rapid neuronal necrosis due to occlusion of cerebral arteries [3, 4]. Subsequently, several cellular signaling cascades in cerebral tissue are altered in the ischemic state, which result in significant aggravation of brain damage in many cases. Due to the inability of neurons to regenerate, neuronal necrosis often produces many permanent neurological sequelae including paralysis, aphasia, coma, and death. Despite biological and technological advances in the field of cerebrovascular research, the recombinant tissue plasminogen activator (rtPA) remains the most effective FDA-approved treatment. Fibrinolytic therapy is administered intravenously within 4.5 hours of symptom onset and is intended to precipitate intravascular clot-retrieval and occluded vessel reperfusion [2, 5, 6]. Though rtPA is effective, many patients are still subject to significant injury as a result of the narrow therapeutic time window, rehemorrhagic complications, and reperfusion injury [7, 8]. Recent advances in endovascular therapy with new generation stent-retrievers and expanded intervention time windows have achieved higher rates of revascularization for patients with large vessel occlusion acute ischemic stroke [9, 10]. However, only 46% of patients treated with endovascular therapy for anterior circulation large vessel occlusion (LVO) achieve functional independence at 90 days with a 15.3% mortality rate [9, 11]. Thus, neuroprotective agents with broad therapeutic windows are urgently needed. A heightened focus on neuroprotection following reperfusion is warranted to reduce disability, morbidity, and mortality following ischemic brain injury.

Autophagy is a highly conserved lysosome-dependent process that maintains cellular homeostasis. It sequesters aging proteins and misfolded molecules for degradation either nonspecifically or by targeting specific protein aggregates. Based on the method and size of cargo delivery to the lysosome, autophagy can be divided into three types: macroautophagy, microautophagy, and chaperone-mediated autophagy (CMA) [12]. In this review, our emphasis will be on macroautophagy. Macroautophagy uses a double membrane-bound vesicle to deliver cytoplasmic cargo to the lysosome, to which it subsequently fuses with to form an autolysosome, leading to digestion [13, 14]. In contrast, microautophagy occurs when intracellular contents are taken up directly by the lysosome, while CMA employs targeted proteins, which are taken up by transmembrane complexes in the lysosome. The process of macroautophagy consists of five stages. Induction is the first stage and is triggered by cellular stress such as endoplasmic reticulum (ER) stress and hypoxia/anoxia. In mammalian systems, autophagy is thought to originate in omegasomes, a monolayer membrane. The omegasome expands and bends into a double-membrane structure called the phagophore, which then engulfs cellular macromolecules and organelles. The double-membraned structure first surrounds intracellular contents to form the autophagosome and then fuses with a lysosome to form the autolysosome in a process called phagophore nucleation. In the next two stages, phagophore expansion and phagophore elongation, the phagophore expands and elongates, respectively. Subsequently, maturation of the phagosome occurs via the acquisition of proteins that are trafficked to the lysosome. The nonessential or damaged constituent contents are then digested by the lysosome and recycled to produce substrates for the maintenance of cytoplasmic balance [14–16]. The process is regulated by a group of autophagy-related genes (ATG) that activate an adaptive response to cellular stressors for the purpose of maintaining cellular energetic homeostasis.

An increasing amount of studies suggest that autophagy activity changes under cerebral ischemia/reperfusion (I/R), while the effect of autophagy on stroke is disputable. The activation of autophagy during ischemia has been demonstrated with electron microscopy and Western blot [17]. Immunofluorescence analysis of the microtubule-associated protein 1A/1B-light chain 3 (LC3), an autophagy-associated protein, also has confirmed the activation of autophagy in ischemic rats [18]. A study by Wen et al. found that autophagy inhibitors, 3-methyladenine (3-MA) and bafilomycin (BFA), reduced infarct volume and motor defects when administered before the onset of ischemia. The study also suggested that excessive autophagy after ischemia lead to cell death, possibly encouraging apoptosis by downregulating B cell lymphoma-2 (Bcl-2) [17]. Interestingly, there are reports that activating autophagy through different signaling pathways could alleviate the infarction outcome [1, 19]. Although there have been conflicting findings regarding the effect of autophagy in ischemia, current studies show that many interventions can improve ischemic injury by regulating autophagy. The present review aims to discuss the recent progress of studies on the molecular mechanisms of autophagy after stroke and the potentials of how autophagy serves as a novel target for neuroprotection to mitigate poststroke cerebral injury.

## 2. Signaling Pathways of Autophagy in Stroke

**Figure 1:**
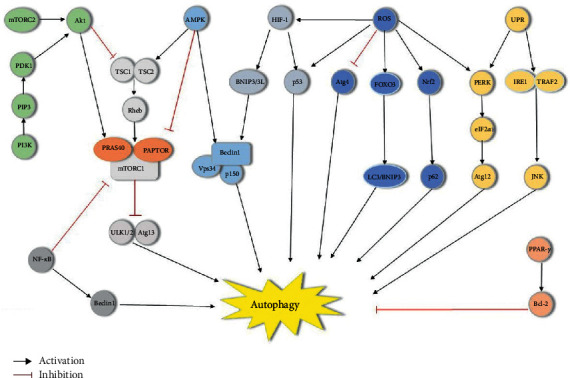
Signaling pathways of autophagy. PI3K produces PIP3 after stimulation. PIP3 forms a docking site containing proteins PDK1 and Akt. When Akt is activated, TSC2 dissociates from TSC1, which improves GTP-Rheb activity and subsequently increases mTORC1 activity. Akt also activates mTORC1 by regulating PRAS40 activity. mTORC1 inhibits ULK1/2 complex activation, causing a decrease in autophagosomes. AMPK regulates the TSC2/TSC1 complex, activating autophagy in contrast to Akt. AMPK also phosphorylates RAPTOR to inhibit mTORC1 activity. AMPK is involved in the regulation of class III PI3K complexes. It modifies VPS34 affinity and phosphorylates Beclin 1 to promote autophagosome formation. HIF-1 can mediate BNIP3/BNIP3L to promote the separation of Beclin 1 from Bcl-2, thus allowing Beclin 1 to participate in autophagosome formation. HIF-1 is involved in the regulation of autophagy through ROS activation. In contrast, PPAR-*γ* enhances Bcl-2 overexpression and inhibits autophagy occurrences. In addition, HIF-1 and ROS can also activate autophagy by regulating the expression of tumor protein p53. NF-*κ*B activates autophagy by inhibiting mTOR activity and promoting Beclin 1 expression. UPR initiated by endoplasmic reticulum stress can mediate autophagy through the PERK/eIF2*α* and Ire1/TRAF2/JNK signaling pathways. ROS can also regulate autophagy through the ER stress-mediated PERK pathway. Furthermore, accumulated ROS can induce Nrf2 and FOXO3 expression, subsequently activating p62 and LC3/BNIP3, respectively. Atg4 is regulated by ROS to promote autophagosome formation.

Postischemic disturbances in the internal environment lead to changes in autophagy activity via the regulation of certain signaling pathways. Ischemia in brain tissue leads to decreased ATP production, oxidative stress, endoplasmic reticulum stress, and calcium overload. Factors regulate autophagy through different pathways [20] and will be discussed as follows.  Figure 1 briefly describes the signaling pathway of autophagy after ischemic stroke.

### 2.1. Mammalian Target of Rapamycin (mTOR) Pathway

mTOR, a protein with serine/threonine kinase activity, coordinates anabolic and catabolic processes to maintain essential homeostasis. It consists of two complexes, mTORC1 and mTORC2, which regulate different aspects of cellular homeostasis [21]. mTORC1 is highly sensitive to rapamycin (specific mTOR inhibitor), promotes anabolic metabolism, and inhibits catabolic processes by inhibiting autophagy. mTORC2 is involved in other distinct signaling complexes and has demonstrated Akt expression promotion [22].

mTOR regulates autophagy activity in ischemia and reperfusion via two major pathways: the Akt-mTOR pathway and the AMPK-mTOR pathway. The Akt-mTOR pathway is modulated by mammalian phosphatidyl-inositol 3-kinase (PI3K) enzymes, which consist of three groups: class I, class II, and class III. Class I PI3Ks are specifically involved in the activation of Akt [23]. Various intracellular and extracellular stimuli activate PI3K, initiating a series of downstream cascades. One of these cascades leads to phosphatidylinositol-3,4,5-trisphosphate (PIP_3_) production, which forms a docking site containing proteins PDK1 and Akt [24]. Akt becomes fully activated after it is phosphorylated by PDK1 and mTORC2 at two amino acid residues (Thr308 and Ser473), respectively. Akt activation then inhibits TSC2 (tuberin), inducing its dissociation from TSC1 (hamartin). When in a complex, TSC1/2 convert GTP-Rheb into GDP-Rheb. Thus, the dissociation of TSC1 and TSC2 improves GTP-Rheb activity and subsequently increases the activity of mTORC1 [5, 22, 24, 25]. Following activation, mTORC1 phosphorylates downstream effector proteins and inhibits formation of the ULK1/2 complex, which is necessary in the early steps of autophagy to generate the autophagosome [20, 26]. Taken together, downregulation of the Akt-mTOR pathway may induce autophagy.

On the contrary, elevations of the AMP/ATP ratio and increased calcium influx as a result of ischemic stress enhance AMPK activity [27, 28]. Once activated, AMPK modulates energy balance by stimulating metabolic processes and inhibiting synthetic processes [29]. It stimulates autophagy through the phosphorylation of specific autophagy-initiating protein complexes and is considered an upstream mediator, though its regulation is complicated and cross-reactive [30]. AMPK phosphorylates TSC2 at Thr1227 and Ser1345 residues, which activates TSC2, and inhibits the separation of the TSC1/TSC2 complex. mTORC1 activity is reduced as a result. Furthermore, AMPK inactivates mTORC1 by phosphorylating RAPTOR, a unique complex found in mTORC1 but not in mTORC2, at Ser722 and Ser792 residues. Since mTORC1 inactivates autophagy-inducing proteins ULK1/2 and ATG13, a decrease in mTORC1 activity may augment ULK1 activation and thus promote autophagy flux [31, 32]. In addition to regulating autophagy through mTOR, AMPK is involved in the regulation of class III PI3K complexes. Another complex, consisting of PIK3C3/VPS34, PIK3R4/p150, and Beclin 1, is encouraged by AMPK in its induction of autophagy formation. AMPK, for example, may modify VPS34's affinity for the other components of the complex to regulate the activity of autophagy. Furthermore, AMPK phosphorylation of Beclin 1 at diverse sites (Thr388, Ser91, and Ser94) can promote autophagosome formation under nutrient-deficiency conditions [33, 34].

### 2.2. Hypoxia-Inducible Factor-1 (HIF-1) Pathway

HIF-1 is a transcription factor that regulates adaptive responses to hypoxic environments, which consists of two subunits: HIF-1*α* and HIF-1*β*. It has become a major focus of neuroscience research since it regulates postischemic pathological processes such as apoptosis, energy metabolism, and gene transcription. Additionally, recent literature indicates that HIF-1 is involved in the regulation of autophagy after stroke [35]. Adenovirus (E1B) 19KD-interacting protein 3(BINP3)/BNIP3-like (BNIP3L), a target gene of HIF-1, is an autophagy inducer. Activated HIF-1 promotes the expression of BINP3 under ischemic conditions. BNIP3/BNIP3L competes with Beclin 1 and dissociates it from the Beclin 1/Bcl-2 complex, stimulating Beclin 1 to participate in the formation of the autophagosome [36]. Lu et al. demonstrated that hypoxic preconditioning (HPC) activated autophagy through the HIF-1/BNIP3/Beclin 1 signaling pathway in SH-SY5Y cells after oxygen and glucose deprivation/reoxygenation (OGD/R). This phenomenon could be reversed by the application of YC-1, a HIF-1 inhibitor. This suggests that HIF is involved in autophagy activation and is a potential therapeutic target for autophagy [37]. Furthermore, BNIP3 can also inhibit Rheb, an upstream activator of mTOR, to promote autophagy. HIF-1 expression is also involved in upregulating mitochondrial autophagy by inhibiting the mTOR signaling pathway, although whether this effect is regulated by BNIP3 needs further verification [38]. Moreover, HIF-1 may activate autophagy by regulating the expression of tumor protein p53 [20].

### 2.3. Unfolded Protein Response (UPR) Signaling Pathways

The endoplasmic reticulum (ER) plays an important role in maintaining intracellular Ca^2+^ balance and synthesizing proteins. During ischemic conditions, misfolded proteins accumulate and Ca^2+^ balance is disrupted, leading to ER stress and initiation of a self-protecting event called the unfolded protein response (UPR) [14]. Autophagy is activated by UPR through the PERK/eIF2*α* and Ire1/TRAF2/JNK signaling pathways [39]. UPR upregulates protein kinase RNA-like ER kinase (PERK) and inositol requiring kinase 1(Ire1) [40]. PERK promotes the expression of Atg12 by eukaryotic initiation factor 2*α* (eIF2*α*) phosphorylation. Atg12 plays an important role in autophagosome formation. Gene knockout of PERK inhibits autophagy, demonstrating that the PERK-eIF2*α* signaling pathway upregulates autophagy [41]. In addition, IRE1 can combine with tumor necrosis factor receptor-associated factor-2 (TRAF-2) and further phosphorylate JNK, thus triggering autophagy [42]. Moreover, activating transcription factor 6 (ATF6), one of the signal transduction pathways of UPR, has been reported to modulate the occurrence of autophagy in stroke, but the specific signaling mechanism is still puzzling [43].

### 2.4. Reactive Oxygen Species (ROS) Pathway

Oxidative stress after ischemic injury causes excessive accumulation of ROS [44], which is related to autophagy regulation [45]. ROS mediates autophagy mainly through intracellular transcription regulation [14]. Elevated ROS increases p53 levels, which activates two regulators of autophagy, Tp53-induced glycolysis and apoptosis regulator (TIGAR), and DNA damage-regulated autophagy modulator (DRAM) [46, 47]. ROS also enhances the transcription of nuclear factor- (erythroid-derived2-) like2 (Nrf2). Subsequently, Nrf2 promotes the expression of autophagy-associated protein p62 to mediate autophagy [48]. ROS stimulates forkhead box O3 (FOXO3) expression to regulate autophagy through activating LC3 and BNIP3 [49]. Furthermore, PERK is also activated by ROS in addition to UPR in its involvement of autophagy regulation [50]. HIF-1 expression is induced by accumulated ROS and stimulates autophagy through the BNIP3/BNIP3L pathway as mentioned above [36, 51]. On the other hand, Atg4, which is responsible for autophagosome membrane elongation, was also shown to be inhibited by ROS, thus supporting autophagosome formation [52]. Atg4 participates in the formation of LC3-I by exposing residues at the C terminus of LC3. Then, LC3-I combines with phosphoethanolamine (PE) to form LC3-II. Moreover, Atg4 is involved in cleavage of LC3-II [13]. It is reported that Atg4 protease activity was inhibited by ROS oxidation [53]. Inhibition of Atg4 may promote autophagy by reducing cleavage of LC3-II [45].

### 2.5. Additional Signaling Pathways

As mentioned previously, HIF-1 binds Bcl-2 in competition with Beclin 1 and releases Beclin 1 to induce autophagy. Unlike HIF-1, peroxisome proliferators-activated receptors (PPAR-*γ*) upregulate Bcl-2 expression to inhibit Beclin 1-mediated autophagy activation [20]. Nuclear factor kappa B (NF-*κ*B), a sensitive transcription factor, has been reported to be involved in autophagy and apoptosis regulation [54]. NF-*κ*B activates autophagy by triggering the expression of protein Beclin 1 [55, 56]. In addition, p50, a subunit of NF-*κ*B, has been reported to inhibit mTOR activity in ischemic stroke mice models [57]. Inhibiting NF-*κ*B and its downstream effector p53 downregulates autophagy and apoptosis, consequently alleviating ischemia/reperfusion injury [58].

## 3. Autophagy in Stroke

Autophagy plays an integral role in the physiological and pathological processes of ischemic stroke through several pathways. Dozens of studies suggest that autophagy is activated after ischemia/reperfusion [17, 20, 59]. Yan et al. demonstrated autophagy activation by detecting the number of autophagosomes using electron microscopy. They observed that the LC3 protein levels in the ischemic penumbra of the cerebral cortex of mice increased between 3 to 24 hours following MCAO reperfusion [59]. Similarly, another study found that LC3 expression increased significantly in the ischemic penumbra after 1 hour of ischemia and continued for 5 hours without reperfusion [60]. Interestingly, the apoptotic protein cleaved caspase-3 was also elevated, and its dynamic changes were similar to those of autophagy, suggesting the coexistence of autophagy and apoptosis in the cerebral ischemic penumbra [61]. However, whether the activation of autophagy promotes neuronal survival or death has been debated. Some studies suggest that induced autophagy after stroke provides a source of energy for cell survival by degrading damaged material. Others suggest that overactivated autophagy may aggravate stroke injury by damaging normal cells and causing autophagic cell death (type II programmed cell death) in addition to apoptosis.

### 3.1. Beneficial Effect of Autophagy in Ischemic State after Stroke

Changes in the intracellular environment, such as mitochondrial dysfunction, oxidative stress, endoplasmic reticulum stress, and apoptosis, may aggravate neurological dysfunction after ischemia and reperfusion. Apoptosis is a type I programmed cell death that is tightly regulated. Proapoptotic genes are activated in ischemic conditions to initiate the apoptotic pathway. Zhang et al. discovered that astragaloside IV could decrease neuronal apoptosis by activating autophagy in HT22 cells after OGD/R [62]. In addition, ezetimibe has proved to alleviate infarct volume and neurobehavioral deficits in middle cerebral artery occlusion (MCAO) rats. The neuroprotective and antiapoptotic effects of ezetimibe were diminished after intervention with the autophagy inhibitor 3-MA [63]. These data suggest that activation of autophagy after ischemia and reperfusion can reduce neuronal damage by decreasing apoptosis. Rapamycin induces autophagy by inhibiting mTOR. Wu et al. found that rapamycin decreased infarction volumes and improved neurologic deficits [64]. It has been reported that autophagy can improve the internal conditions of the cell after ischemia by removing damaged mitochondria [65]. Li et al. also found that rapamycin-activated autophagy improved mitochondrial function and alleviated ischemic injury, and that protective effect was reversed by 3-MA [66].

### 3.2. Autophagy Negatively Affects Stroke Outcome

Accumulating results have suggested that autophagy activation aggravates neurological dysfunction after ischemia and reperfusion. Intraperitoneal injection of 3-MA reduced ischemic nerve injury and brain edema in permanent middle cerebral artery ischemia models [17]. Treatment with tetrahydroxystilbene glucoside (TSG), one of the essence of the *Fallopia multiflora*, also reduced infarct volume and neurobehavioral deficits in ischemia/reperfusion mice by inhibiting autophagy [67]. Zhang et al. studied the TP53-induced glycolysis and apoptosis regulator (TIGAR), which functions as a fructose-2,6-biphosphatase, to verify the role of autophagy activation after ischemia-reperfusion. They found that autophagy decreased in TIGAR-transgenic mice, as opposed to TIGAR-knockout mice. Knockout of TIGAR not only elevated autophagy but also increased infract volume and neurological deficit scores. These detrimental effects were blocked by treatment with 3-MA after ischemia/reperfusion. Thus, the neuroprotective effect of TIGAR was produced in part by inhibiting autophagy [68]. Puerarin, a traditional Chinese herb, has been demonstrated to alleviate brain dysfunction after ischemia/reperfusion by depressing autophagy protein expression via the AMPK-mTOR-ULK1 signaling pathway [8]. Similarly, Jiang and his colleagues confirmed that inhibiting excessive autophagy can reduce postischemia-reperfusion damage [69]. Luo et al. discovered that the use of dexmedetomidine could significantly reduce brain injury after ischemic stroke by inhibiting autophagy. The effect of dexmedetomidine was enhanced by 3-MA and diminished by rapamycin [70]. These data suggest that autophagy inhibition is neuroprotective.

## 4. Autophagy-Mediated Neuroprotection in Ischemia/Reperfusion Injury

Although the current consensus is that autophagy is a double-edged sword after ischemia-reperfusion, a number of neuroprotective strategies targeting neuronal autophagy have been discovered, including neuroprotective drugs, ischemic preconditioning, electroacupuncture, hyperbaric oxygen preconditioning, and nucleic acid therapies. These are summarized in Table 1.

### 4.1. Neuroprotective Agents on Autophagy

Today, pharmacological development remains the primary focus of ischemic stroke research. As mentioned above, ezetimibe attenuates neuronal apoptosis via autophagy activation after MCAO in rats [63]. Ezetimibe administration decreased infarct volumes, neurological deficits, and cerebral cholesterol levels at 24 hours after MCAO. They also noted that Beclin 1 immunopositive cells increased in ischemic rats, while 3-MA reversed the neuroprotection provided by ezetimibe. Traditional Chinese medicines have been shown to have therapeutic effects by regulating autophagy. Puerarin has proved to alleviate brain dysfunction after ischemia/reperfusion by depressing autophagy protein expression via the AMPK-mTOR-ULK1 signaling pathway [8]. Ginkogolide K (GK) pretreatment has been reported to induce autophagy under ischemia, promoting astrocyte proliferation and migration after reoxygenation [71]. Ginsenoside Rb1 (GRb1) ameliorates brain damage and increases autophagy after ischemia/reperfusion [72]. Similarly, triptolide treatment was reported to decrease apoptosis following ischemia, in association with induced autophagy [73]. In another study, rapamycin showed potential neuroprotective effects in both permanent middle cerebral artery ligation (pMCAL) and embolic clot middle cerebral artery occlusion (eMCAO). Infarct volumes measured via TTC staining after eMCAO and pMCAL indicated rapamycin-reduced injury lesions and upregulated autophagy [64, 66, 74]. Moreover, metformin is reported to lessen the risk of stroke by enhancing autophagy [19], while nicotinamide phosphoribosyl transferase (NAMPT) promotes cell survival by regulating autophagy after cerebral ischemia [75]. Overall, conferring neuroprotection through targeting autophagy with drug therapy is feasible.

### 4.2. Nondrug Therapies on Autophagy

#### 4.2.1. Electroacupuncture (EA)

EA was reported to be protective against cerebral ischemic injury [91, 92]. Recently, it was observed that EA treatment reduces cell apoptosis after ischemia [93, 94]. The neuroprotective effect of EA and its relation to autophagy have been assessed by Huang et al. [76]. EA is involved in the autophagy initiation, vesicle nucleation, and autophagosome maturation, in addition to autophagolysosome degradation. It was also shown that EA treatment influences autophagy flux by regulating the expression of autophagy-related proteins including the ULK1 complex, Beclin 1, and mTOR [77, 78]. However, it is unclear whether EA promotes or suppresses autophagy. In addition, different EA parameters such as the selection of acupoints, duration of stimulation, and the timing of ischemia/reperfusion have different effects against neuronal ischemic/reperfusion injury.

#### 4.2.2. Ischemic Preconditioning and Postconditioning (IPC)

IPC confers neuroprotection by increasing the tolerance to fatal ischemic exposure, which has been shown to be associated with autophagy [95, 96]. The results of Sheng's group showed that the number of autophagosomes in neurons increased with the induction of LC3 immunopositivity in the ischemic preconditioning model. 3-MA, an autophagy inhibitor, reversed and weakened ischemic preconditioning-induced autophagy activation and protective effects [79]. These findings indicate that enhanced autophagy contributes to neuroprotection induced by ischemic preconditioning, as demonstrated recently [80–82]. Moreover, an in vitro study confirmed that ischemic preconditioning induces autophagy activation through the AMPK pathway [97]. In addition, changes in autophagy activity are implicated in the resistance to cerebral ischemia conferred by remote ischemic postconditioning. Guo et al. found that remote limb ischemic postconditioning (RIPoC) induced autophagy-related protein expressions when compared with the ischemia/reperfusion group. Inhibition of autophagy through pharmacological means not only abolished the effect of conditioning against ischemia but also reversed the antiapoptotic effect [83]. On the other hand, Chen et al. revealed that the protective effect of RIPoC was related to the inhibition of autophagy activation [84]. These contradictory results may be due to the different influences of ischemia time and means of conditioning on autophagy activity in animal models. Taken together, ischemic pre- and postconditioning may induce neuroprotection through the regulation of autophagy.

#### 4.2.3. Hyperbaric Oxygen Administration (HBO)

HBO is an effective method in the treatment of brain trauma in clinical practice. Currently, researchers have found that it also has a therapeutic effect in ischemic injury [98]. Although the potential mechanisms remain unclear, much attention has been given to the relationship between autophagy and HBO [99, 100]. In an MCAO model, autophagy was involved in the tolerance to cerebral ischemia conferred by HBO [60]. In this study, the authors discovered that HBO reduced cerebral damage through the enhancement of autophagy-related proteins LC3-II and Beclin 1. 3-MA reduced the HBO-induced neuroprotective effect, suggesting that activated autophagy is one of the mechanisms of HBO administration. In addition, HBO preserved the integrity of the lysosomal membrane and promoted the formation of autolysosomes in transient focal cerebral ischemia rats. Cystatin C (CysC) is a determinant for neuroprotection in HBO therapy as it promotes cerebral autophagic flux after ischemia [85]. HBO-induced autophagy in cerebral ischemia/reperfusion was also found to be neuroprotective by Wang and colleagues [86]. In contrast, a recent article by Chen et al. reported that HBO conferred neuroprotection by autophagy inhibition [87]. HBO should be further studied as a promising nonpharmacological and noninvasive treatment.

#### 4.2.4. Nucleic Acid Therapies

MicroRNAs (miRNAs) are composed of 20–25 endogenous, noncoding, single-stranded RNA molecules. They regulate target genes' expressions and can modulate cell proliferation, differentiation, apoptosis, and metabolism [101]. Recent studies suggest that cerebral ischemia leads to abnormal changes in miRNA expression levels, which are involved in the etiology and pathology of sequelae after stroke. Dharap et al. demonstrated that 24 miRNAs were increased, while 23 other miRNAs were decreased following stroke [88]. These altered miRNAs may modulate genetic expression, which suggests that miRNA may be a potential therapeutic target to reduce cerebral injury following ischemia/reperfusion. This neuroprotection may be offered through an autophagy-mediated mechanism [90]. Wang et al. found that miR-30a expression was downregulated, while autophagy expression was upregulated in the in vivo and in vitro ischemia/reperfusion model. It has been confirmed that the downregulation of miR-30a abolished ischemia injury by Beclin 1-mediated autophagy. miR-30a negatively regulates Beclin 1 expression through recognizing the 3′-untranslated region (3'UTR) of Beclin 1 [1]. Similarly, miR-30d-5p, which regulates the 3'UTR of Beclin 1, is presumed to promote neuronal death in hypoxic-ischemic (HI) rats by inhibiting autophagy [89]. Chen et al. observed that the suppression of miR-497 increased neuronal autophagy and alleviated ischemia injury, especially in young rats [90]. Taken together, these findings indicate that miRNA expression after stroke may be involved in neuroprotection through autophagy.

## 5. Conclusion

Current studies consistently report elevation of autophagic flux following ischemia/reperfusion. However, the role of autophagy after acute ischemia and reperfusion remains uncertain, especially in regard to the precise functions that mediate cell survival or cell death. Overall, autophagy may act through the “Goldilocks” principle: excessive or inadequate induction of autophagy may be maladaptive, while a specific level of autophagy may be beneficial. Moreover, the beneficial or detrimental effects of autophagy may be dependent upon the severity or length of ischemia. Further research is warranted to assess how autophagy activation is beneficial. There is consensus that signaling pathways associated with autophagy comprise potential therapeutic targets for novel and neuroprotective strategies. Multimodal targeting of autophagy at different time points may serve as an effective method in the treatment of stroke.

## Figures and Tables

**Table 1:** Neurotherapeutics targeting autophagy after I/R.

Interventions	Key findings	References
Pharmacological interventions		
Traditional Chinese medicine (ginsenoside Rb1, puerarin, ginkogolide K, triptolide, and ezetimibe)	(i) Ginsenoside Rb1 ameliorated neuronal injury and increased autophagy after I/R (ii) Puerarin reduced ischemic brain damage by inhibiting autophagy through the AMPK-mTOR-ULK1 signaling pathway (iii) GK pretreatment induced autophagy under ischemia and promoted astrocyte proliferation and migration after reoxygenation (iv) Triptolide decreased apoptosis and induced autophagy, thus conferring neuroprotection (v) Ezetimibe attenuated neuronal apoptosis by activating autophagy and reduced cerebral cholesterol levels	[8] [63] [71] [72] [73]
Rapamycin	(i) Rapamycin reduced infarct sizes and cell death in both permanent MCAL and embolic MCAO (ii) Rapamycin may attenuate stroke injury lesion sizes and upregulate autophagy	[64] [66] [74] [19] [75]
Metformin	(i) Acute metformin preconditioning lessened the risk of stroke by enhancing autophagy	
Nampt	(i) Nampt induced autophagy and promoted cell survival after cerebral ischemia	

Nonpharmacological interventions		
Electroacupuncture	(i) EA is involved in the initiation of autophagy, vesicle nucleation, and maturation of autophagosomes, in addition to the degradation of autophagolysosomes (ii) Treatment with EA may influence autophagy activity through regulating autophagy-related proteins	[76] [77] [78]
Cerebral ischemic pre-/postconditioning or remove ischemic conditioning	(i) Ischemic preconditioning alleviates cerebral I/R injury by activating autophagy, therefore improving neurologic functions (ii) RIPoC decreased infarct volume, neurological deficits, and cell apoptosis after I/R through regulating autophagy	[79] [80] [81] [82] [76] [83] [84]
Hyperbaric oxygen preconditioning (HBO)	(i) HBO preconditioning preserved the integrity of the lysosomal membrane and formed autolysosomes in transient focal cerebral ischemia to activate autophagy (ii) HBO-mediated autophagy after cerebral I/R was also found to be neuroprotective	[85] [86] [87]

Nucleic acid therapies		
miR-497 miR-30d-5p miRNA-30a	(i) Cerebral ischemia can lead to abnormal changes in miRNA expression levels involved in the etiology and pathology of stroke (ii) miR-30a negatively regulates the 3'UTR of Beclin 1 to inhibit autophagy, and miR-30d-5p regulates autophagy in a similar way (iii) Suppression of miR-497 could increase neuronal autophagy activity to alleviate ischemia injury	[1] [88] [89] [90]

## Data Availability

The datasets used and analyzed during the current study are available from the corresponding author on reasonable request.
